# Wireless Sensor Array Network DoA Estimation from Compressed Array Data via Joint Sparse Representation

**DOI:** 10.3390/s16050686

**Published:** 2016-05-23

**Authors:** Kai Yu, Ming Yin, Ji-An Luo, Yingguan Wang, Ming Bao, Yu-Hen Hu, Zhi Wang

**Affiliations:** 1 State Key Laboratory of Industrial Control Technology, Zhejiang University, Hangzhou 310027, China; kaiyuzju@gmail.com (K.Y.); yinming1026@zju.edu.cn (M.Y.); 2 Institute of Information and Control, Hangzhou Dianzi University, Hangzhou 310018, China; luojian@hdu.edu.cn; 3 Shanghai Institute of Microsystem and Information Technology, Chinese Academy of Sciences, Shanghai 200050, China; wyg@mail.sim.ac.cn; 4 Institute of Acoustics, Chinese Academy of Sciences, Beijing 100190, China; baoming@mail.ioa.ac.cn; 5 Department of Electrical and Computer Engineering, University of Wisconsin-Madison, Madison, WI 53706, USA; yhhu@wisc.edu

**Keywords:** wireless sensor network, sensor array, compressive sensing, array processing

## Abstract

A compressive sensing joint sparse representation direction of arrival estimation (CSJSR-DoA) approach is proposed for wireless sensor array networks (WSAN). By exploiting the joint spatial and spectral correlations of acoustic sensor array data, the CSJSR-DoA approach provides reliable DoA estimation using randomly-sampled acoustic sensor data. Since random sampling is performed at remote sensor arrays, less data need to be transmitted over lossy wireless channels to the fusion center (FC), and the expensive source coding operation at sensor nodes can be avoided. To investigate the spatial sparsity, an upper bound of the coherence of incoming sensor signals is derived assuming a linear sensor array configuration. This bound provides a theoretical constraint on the angular separation of acoustic sources to ensure the spatial sparsity of the received acoustic sensor array signals. The Cram$\overset{´}{e}$r–Rao bound of the CSJSR-DoA estimator that quantifies the theoretical DoA estimation performance is also derived. The potential performance of the CSJSR-DoA approach is validated using both simulations and field experiments on a prototype WSAN platform. Compared to existing compressive sensing-based DoA estimation methods, the CSJSR-DoA approach shows significant performance improvement.

## 1. Introduction

Direction of arrival (DoA) estimation using acoustic sensor arrays has attracted significant interest due to its wide applications [1,2,3,4]. Traditionally, DoA is realized using sensor arrays, such as passive towed-array sonar systems, where all sensors are wire-connected to a fusion center (FC) [5]. For wireless sensor array networks (WSAN) [6,7], arrays (locally wire/wireless-connected clusters) of dispersed sensors are deployed over large sensor fields and communicate wirelessly to the FC. However, remote sensor arrays often suffer from restricted resources, such as local power supply, local computational capacity and wireless transmission bandwidth. Therefore, data samples at remote sensor arrays need to be compressed before transmitting to the FC. On the other hand, executing sophisticated data compression algorithms on remote sensors is also restricted by local power consumption and computational ability.

To meet those resource requirements of the WSAN, the newly-emerged compressive sensing (CS) technology [8,9,10] has great potential because of its ability to reconstruct the raw signal from only a small number of random measurements. In this work, we propose a novel DoA estimation approach that accomplishes low power, robust DoA estimation over a WSAN platform, which reduces the volume of transmitted data without complicated local data compression operations. Incorporating a CS-based formulation, this approach is able to directly estimate the incident angles of acoustic sources from randomly-sampled acoustic signals. This is made possible by fully exploiting the joint spatial and spectral sparse structure of the acoustic signals acquired by the sensor array. Hence, we refer to this approach as compressive sensing joint sparse representation direction of arrival (CSJSR-DoA) estimation method.

Existing CS-based source DoA estimation methods can be categorized into two different approaches: compressive bearing estimation (COBE) [11,12,13] and compressive sensing array DoA estimation (CSA-DoA) [14]. With COBE, the incident angles (DoAs) are modeled as a sparse angle vector, with non-zero entries indicating the presence of (a few well separated) acoustic sources. The acoustic signals received at each sensor can then be represented as the product of a redundant steering matrix and a sparse angle vector. The steering matrix, consisting of steering vectors of all possible incident angles, must be estimated using the received noisy acoustic signal at a reference node. Thus, the raw data at the reference node and randomly-projected measurements of the non-reference nodes need to be transmitted to the remote FC. This imposes a heavy communication cost and excessive energy consumption at remote sensor arrays. With CSJSR-DoA, individual sensor nodes only perform data acquisition and random subsampling, while the DoA estimation is performed at the FC. Therefore, no local computation is required at the remote sensor nodes, and the CSJSR-DoA approach is more suitable for a resource-restricted WSAN system.

With the CSA-DoA [15,16,17] method, analog sensor array data are randomly projected onto a lower dimensional subspace in the analog domain before being converted into digital samples using an analog to digital converter (ADC). As such, fewer ADCs are required. This approach is similar to the CS-camera projection and was originally proposed in [18]. The steering vectors of different incident angles at a certain frequency are used to form the reconstruction dictionary. Further work extends this type of array processing method to broadband scenarios [19,20]. Since the data volume reduction is realized in the analog domain, special analog electronic circuits are required to implement the CSA-DoA approach. The proposed CSJSR-DoA approach, on the other hand, requires no special-purpose analog hardware. Some similar work [21] uses the angle domain sparsity of sources and formulates the narrow band signal of an antenna array within the BCS [22,23] framework. However, it is utilized as an alternative DoA estimation approach, and no data compression is considered.

**Figure 1:**
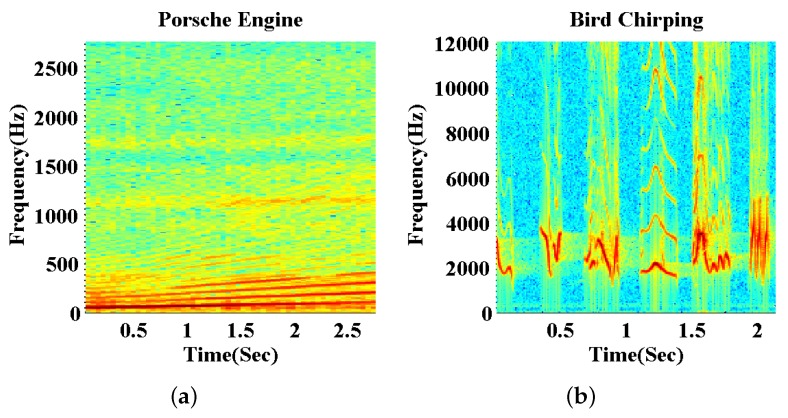
Spectrogram of (**a**) a Porsche engine and (**b**) a bird chirping.

Another distinction between the proposed CSJSR-DoA approach and these existing CS-based DoA estimation approaches is that the CSJSR-DoA approach exploits both the spatial and spectral domain structure of the acoustic sensor array signals. We argue that many practical broadband acoustic signals can be characterized by a few dominant frequency entries. With the purpose of DoA estimation, it would be sufficient to focus on these few dominant frequency entries and to exploit their frequency sparsity to enhance the performance. For example, Figure 1 shows the spectrograms of two types of broadband sources: an engine sound of a Porsche vehicle and a bird chirping [24]. It is clear that these broadband acoustic signals are dominated by multiple dominant frequency entries. The frequency domain sparse structure [25] illustrated in these figures may be utilized to realize efficient compressive sensing.

The main contributions are:

1. A joint sparse representation-based DoA estimation approach is proposed, that exposes the joint spatial and spectral domain sparse structure of array signals and incorporates the multiple measurement vector [26] approach to solve the DoA estimation problem;
2. A theoretical mutual coherence bound of a uniform linear sensor array is provided, which defines the minimum angular separation of the sources that are required for the CSJSR-DoA approach to yield a reliable solution with a high probability;
3. The Cram$\overset{´}{e}$r–Rao bound of the CSJSR-DoA estimator is derived to quantify the theoretical DoA estimation performance;
4. A prototype acoustic WSAN platform is developed to validate the effectiveness of the proposed CSJSR-DoA approach.

The remainder of this paper is organized as follows. In Section 2, the array signal model, the sparsity model of narrowband array processing and the CS theory are briefly reviewed. The CSJSR-DoA approach is derived in Section 3. In Section 4, the theoretical analysis of the DoA estimation performance, as well as the Cram$\overset{´}{e}$r–Rao bound are derived. In Section 5, the performance of the CSJSR-DoA approach is evaluated by simulations and field experiments using a prototype wireless sensor array platform. At last, the conclusion is summarized in Section 6.

## 2. Background

In this paper, the transposition and complex-conjugate transposition operations are denoted by superscripts ${\left(\cdot \right)}^{T}$ and ${\left(\cdot \right)}^{H}$, respectively. Lowercase and uppercase bold symbols denote vectors and matrices, respectively. The major symbols used in this paper are summarized in Table 1.

### 2.1. Signal Model and Joint Spatial-Spectral Sparse Structure

A WSAN consists of one or more sensor arrays and one FC. Sensors within the same sensor array are arbitrarily deployed and connected to the FC via wireless channels. Sensor arrays are battery powered and have limited processing capabilities. The FC is connected to the infrastructure and has stronger processing capabilities. To conserve energy at remote sensor arrays, it is desirable to reduce the volume of data to be transmitted via wireless channels and to move as much of the processing tasks to the FC as possible.

For simplicity of notation, in this work, we assume a single sensor array consisting of *H* acoustic sensor nodes, and it communicates wirelessly via radio channels to a single FC. Each sensor node is equipped with an omni-directional microphone. The received acoustic signals will be sampled at a sampling frequency of $f_{s}$ Hz. *N* samples will be grouped into a frame (a snapshot) from which a DoA estimate will be made. We assume that state-of-the-art wireless synchronization protocols, such as Reference Broadcast Synchronization (RBS) [7], are applied so that clocks at sensor nodes can be synchronized at a precision in the order of micro-seconds, which is sufficiently accurate for acoustic DoA estimation.

We assume there are *Q* targets. The *q*-th source emits an acoustic signal and contains no more than $R_{q}$ ($N>R_{q}$) dominant frequency entries. Denote $s_{q}$ to be a N×1 frame vector of the *q*-th source received at the reference node of the sensor array. Its *N*-point DFT (discrete Fourier transform) may be decomposed into two components:

(1) $$s_{q}=\Psi \left(r_{q}+\nu_{q}\right)$$

where **Ψ** is an N×N inverse DFT matrix, $r_{q}$ is an N×1 and $R_{q}$-sparse vector, containing no more than $R_{q}$ DFT coefficients with larger magnitude, and $\nu_{q}$ contains the remaining smaller DFT coefficients.

The *h*-th sensor node of the same sensor array will receive the same $s_{q}$ with a relative delay $\tau_{h,q}$. Thus, the data received at the *h*-th sensor are the time-delayed summation of *Q* sources. That is:

(2) $$x_{h}\left(t\right)=\sum_{q=1}^{Q}s_{q}\left(t-\tau_{h,q}\right)+\omega_{h}\left(t\right)$$

where $\omega_{h}\left(t\right)$ is the zero mean white Gaussian noise at the *h*-th sensor.

**Figure 2:**
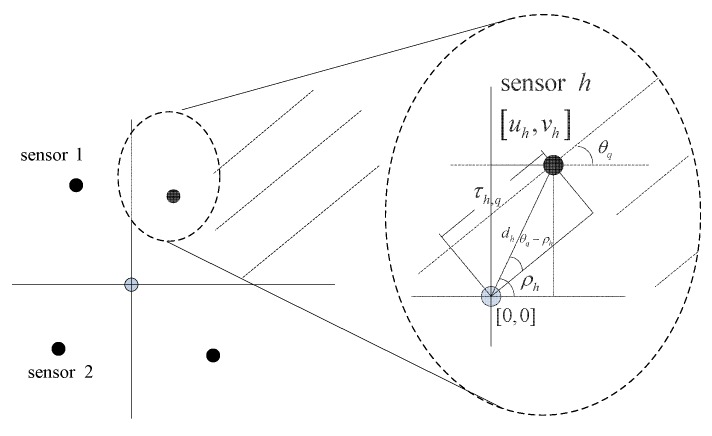
Array time delay model.

Figure 2 is the time delay model of a sensor array. Define $\theta_{q}$ as the incident angle of the *q*-th source, *c* as the speed of acoustic signal and $\left[u_{h},v_{h}\right]$ as the position of the *h*-th sensor with respect to the array centroid ($\sum_{h=1}^{H}\left[u_{h},v_{h}\right]=\left[0,0\right]$). The time delay $\tau_{h,q}$ is given by:

(3) $$\tau_{h,q}=d_{h}\cos\left(\theta_{q}-\rho_{h}\right)/c=d_{h}\left[\cos\left(\theta_{q}\right)\cos\left(\rho_{h}\right)+\sin\left(\theta_{q}\right)\sin\left(\rho_{h}\right)\right]/c=\frac{1}{c}\left(\cos\left(\theta_{q}\right)u_{h}+\sin\left(\theta_{q}\right)v_{h}\right)$$

where $\rho_{h}$ is the direction of the *h*-th sensor with respect to the array centroid. After exploiting *N*-point DFT to $x_{h}={\left[x_{h}\left(1\right),\cdots ,x_{h}\left(N\right)\right]}^{T}$, its frequency domain expression is given by:

(4) $${\tilde{d}}_{h}=\Psi^{-1}x_{h}$$

where ${\tilde{d}}_{h}={\left[d_{h}\left(0\right),d_{h}\left(1\right),\cdots ,d_{h}\left(N-1\right)\right]}^{T}$, and each component $d_{h}\left(k\right)$ is equivalent to multiplying the *k*-th entry of the acoustic spectrum $r_{q}+\nu_{q}$ by a phase shift $\exp(-j\omega_{k}\tau_{h,q})$. That is:

(5) $$d_{h}\left(k\right)=\sum_{q=1}^{Q}\exp\left(-j\omega_{k}\tau_{h,q}\right)\left(r_{q}\left(k\right)+\nu_{q}\left(k\right)\right)+w_{h}\left(k\right)$$

where $w_{h}\left(k\right)$ is the frequency domain expression of $\omega_{h}\left(t\right)$. Since $x_{h}$ consists of all acoustic signals, the N×1 vector ${\tilde{d}}_{h}$ will be *R*-sparse ($\max\left(R_{q}\right)\leq R\leq \sum_{q=1}^{Q}R_{q}$). This is because the dominant components of *Q* sources may fall within overlapped or different spectrum bands.

Consider the array data spectrum $d\left(k\right)={\left[d_{1}\left(k\right),d_{2}\left(k\right),\cdots ,d_{H}\left(k\right)\right]}^{T}$ of *H* sensors at the *k*-th frequency; one may write:

(6) $$d\left(k\right)=\sum_{q=1}^{Q}a_{q}\left(k\right)r_{q}\left(k\right)+w\left(k\right)$$

where $a_{q}\left(k\right)={\left[\exp\left(-j\omega_{k}\tau_{1,q}\right),\cdots ,\exp\left(-j\omega_{k}\tau_{H,q}\right)\right]}^{T}$ is the steering vector of the *q*-th source, $w\left(k\right)=[w_{1}^{\prime }\left(k\right),w_{2}^{\prime }\left(k\right),\cdots$$,w_{H}^{\prime }{\left(k\right)]}^{T}$, $w_{h}^{\prime }\left(k\right)=\sum_{q=1}^{Q}\exp\left(-j\omega_{k}\tau_{h,q}\right)\nu_{q}\left(k\right)+w_{h}\left(k\right)$.

The relative delay $\tau_{h,q}$ is the time for the acoustic wave of the *q*-th source traveling from the array centroid to the *h*-th node in the array along the incident angle $\theta_{q}$. Suppose that the entire range of incident angles is divided into L(≫Q) divisions with each division corresponding to a quantized incident angle $\theta_{\ell }=\ell /L$, 0≤ℓ≤L-1. We can construct a redundant H×L *steering matrix* $A_{L}\left(k\right)=\left[a_{1}\left(k\right),a_{2}\left(k\right),\cdots ,a_{L}\left(k\right)\right]$ that includes *L* steering vectors. Here, $a_{\ell }\left(k\right)={\left[\exp\left(-j\omega_{k}\tau_{1,\ell }\right),\exp\left(-j\omega_{k}\tau_{2,\ell }\right),\cdots ,\exp\left(-j\omega_{k}\tau_{H,\ell }\right)\right]}^{T}$ is defined as the *steering vector* corresponding to an incident angle $\theta_{\ell }$. Furthermore, define a L×1, *Q*-sparse source energy vector r(k) such that:

(7) $$r_{\ell }\left(k\right)=\left\{\begin{array}{cc} r_{q}\left(k\right) & \theta_{\ell }\leq \theta_{q}\leq \theta_{\ell +1}\\ 0 & \mathrm{otherwise}. \end{array}\right.$$

If *L* is sufficiently large, it is safe to assume that different $\theta_{q}$ will fall into different angle bins. With this change of notation, Equation (6) can be rewritten as:

(8) $$d\left(k\right)=A_{L}\left(k\right)r\left(k\right)+w\left(k\right)$$

where $r\left(k\right)={\left[r_{1}\left(k\right),r_{2}\left(k\right),\cdots ,r_{L}\left(k\right)\right]}^{T}$. With Equations (4) and (6), one may construct an H×N matrix:

(9) $$D=\left[d(0),d(1),\cdots ,d(N-1)\right]={\left[{\tilde{d}}_{1},{\tilde{d}}_{2},\cdots ,{\tilde{d}}_{H}\right]}^{T}$$

By performing row-vectorization of matrix D, we have an H·N×1 vector:

(10) $$\tilde{d}={\left[{\tilde{d}}_{1}^{T},{\tilde{d}}_{2}^{T},\cdots ,{\tilde{d}}_{H}^{T}\right]}^{T}$$

Similarly, by performing column-vectorization of matrix D, we have another H·N×1 vector:

(11) $$\bar{d}={\left[d^{T}\left(0\right),d^{T}\left(1\right),\cdots ,d^{T}\left(N-1\right)\right]}^{T}$$

Since the entries of both $\tilde{d}$ and $\bar{d}$ are associated with the same D matrix, there exists an H·N×H·N permutation matrix M such that:

(12) $$\tilde{d}=M\bar{d}$$

Equation (12) provides a link between $\tilde{d}$, which exhibits the *R*-sparsity in the frequency domain, and $\bar{d}$, which exploits the *Q*-sparsity in the spatial (incident angle) domain. In other words, the array samples, as summarized in matrix D, exhibit a joint frequency and spatial (angle) domain sparsity that will be exploited later in this paper.

### 2.2. Compressive Sensing and Random Sub-Sampled Measurements

The presence of sparsity, as described in the models above, promises great potential for introducing CS to reduce the amount of transmitted sensor data.

The compressive sensing theory [27] states that a signal x may be reconstructed perfectly from a dimension reduced measurement $y\in R^{M\times 1}$ through a linear measurement system y=Φx+n when $\alpha =\Psi^{-1}x$ is a *K*-sparse vector. This is often accomplished by solving a constrained optimization problem:

(13) $${\min\;\;||\alpha ||}_{1}\;\;\;s.t.\;\;||\;y-\Phi \Psi \alpha ||<\sigma$$

where ${\left||\alpha |\right|}_{1}$ is the $\ell_{1}$ norm of vector α, **Φ** is the measurement matrix, **Ψ** is the sparse matrix and σ is a noise threshold. To confirm stable reconstruction, the measurement matrix **Φ** is usually chosen to be a random Gaussian matrix or a random binary matrix.

The measurement vector in traditional compressive sensing problem settings is often computed as a weighted linear combination of the observed high dimensional signals. For example, in **COBE** [13], the N×1 sensor data vector in a frame will be multiplied to an M×N measurement matrix (M<N) digitally to yield a measurement vector **y** and then transmitted via a wireless channel to the FC. This obviously requires much computation and is likely to further deplete the energy reserve on sensor nodes. In **CSA-DoA** [15], an analog filter needs to be inserted before the ADC to realize the measurement operation. This approach requires special-purpose hardware and can be quite expensive.

In this work, time domain sensor data will be purposely discarded and will not be transmitted to the FC. By doing so, data reduction may be achieved without incurring expensive compression computations on the sensor nodes. Specifically, we will use a random sub-sampling matrix [28] defined on a set of non-uniform, yet known random sampling intervals $\left\{\tau_{m};1\leq m\leq M\right\}$. Let a sequence be u={u(1),u(2),⋯,u(M)} such that u(1)=1, and:

(14) $$u\left(m\right)=u\left(m-1\right)+r\left(\tau_{m}\right),\;2\leq m\leq M$$

where r(·) is the rounding operation. Then, the (m,n)-th element of this proposed random subsampling matrix is given by:

(15) $$\begin{array}{c} \Phi (m,n)=\left\{\begin{array}{cc} 1 & 1\leq n=u(m)\leq N\\ 0 & \mathrm{otherwise} \end{array}\right. \end{array}$$

The selection of the proposed random subsampling matrix is because the product of ΦΨ is a partial Fourier matrix, which has been proven to satisfy the restricted isometry property (RIP) property with a high probability [29].

Furthermore, in this work, the lossy nature of wireless channels will be exploited by modeling the packet loss during wireless transmission over noisy channels as a form of (involuntary) random data sub-sampling. It is well known that due to different link types (amplify forward and direct link) [30], power allocation [31] and time-varying channel conditions, wireless transmissions are likely to suffer from packet loss, and the identities of lost data packets are known at the FC. Hence, this type of packet loss can be modeled as another form of random sub-sampling. In our recent work [32], the data packet loss is modeled with a *random selection matrix*.

In this work, both the random sub-sampling matrix and the random selection matrix will be incorporated as a combined measurement matrix that requires no computation on the sensor nodes while achieving the desired data reduction. Denote the random sampling matrix at the *h*-th sensor node as $\Phi_{h}$ and the random selection matrix over the wireless channels between the *h*-th sensor node and the FC as $\Phi_{loss}^{h}$. The combined equivalent measurement matrix may then be obtained as:

(16) $$\Phi_{e}^{h}=\Phi_{loss}^{h}\cdot \Phi_{h}.$$

## 3. CSJSR-DoA Formulation

**Figure 3:**
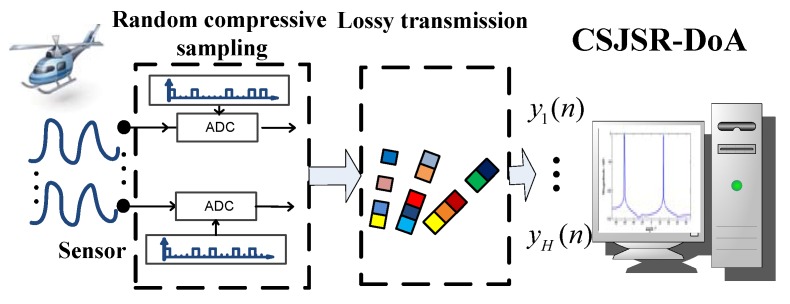
Schematic diagram of the CSJSR-DoA approach.

The proposed CSJSR-DoA algorithm consists of three major steps: (1) at the *h*-th wireless sensor node, the received and digitized acoustic signal $x_{h}$ will be sub-sampled using random compressive sampling as described in Equation (15) to yield a node measurement vector $\Phi_{h}x_{h}$; (2) these node measurement vectors will then be transmitted through lossy wireless channels to the FC; (3) The FC will receive an overall measurement vector $y={\left[y_{1}^{T},y_{2}^{T},\cdots ,y_{H}^{T}\right]}^{T}$, which will then be processed by the CSJSR-DoA approach to directly estimate a sparse DoA indicator vector. The schematic diagram of the proposed CSJSR-DOA approach is summarized in Figure 3.

Note that at the FC, the received compressive measurement of the *h*-th sensor node may be expressed as:

(17) $$y_{h}=\Phi_{loss}\cdot \Phi_{h}x_{h}=\Phi_{e}^{h}\Psi {\tilde{d}}_{h}$$

Hence, the overall measurement vector y may be expressed as:

(18) $$\left[\;\begin{array}{c} y_{1}\\ y_{2}\\ \vdots \\ y_{H} \end{array}\;\right]\;=\;\left[\;\begin{array}{cccc} \;\Phi_{e}^{1}\Psi \; & & & \\ & \;\Phi_{e}^{2}\Psi \; & & \\ & & \;\ddots \; & \\ & & & \;\Phi_{e}^{H}\Psi \; \end{array}\;\right]\;\;\left[\;\begin{array}{c} {\tilde{d}}_{1}\\ {\tilde{d}}_{2}\\ \vdots \\ {\tilde{d}}_{H} \end{array}\;\right]$$

or in matrix form,

(19) $$y=\Theta \tilde{d}$$

where $\Theta =\mathrm{diag}(\Phi_{e}^{1}\Psi ,\Phi_{e}^{2}\Psi ,\cdots ,\Phi_{e}^{H}\Psi )$ is the joint sparse representation matrix. As discussed earlier, $\left\{{\tilde{d}}_{h},1\leq h\leq H\right\}$ shares a common *R*-sparse structure, since the signal measured at one sensor node would be a time-delayed version of those at other sensors.

Next, from Equations (8) and (11), one may write:

(20) $$\bar{d}=A_{L}r_{L}+w,$$

where $r_{L}={\left[r_{L}{\left(0\right)}^{T},r_{L}{\left(1\right)}^{T},\cdots ,r_{L}{\left(N-1\right)}^{T}\right]}^{T}$, $w={\left[w{\left(0\right)}^{T},w{\left(1\right)}^{T},\cdots ,w{\left(N-1\right)}^{T}\right]}^{T}$, $A_{L}$ is the HN×NL joint steering matrix:

$$A_{L}=\left[\;\begin{array}{cccc} \;A_{L}\left(0\right)\;\; & \;\; & \;\; & \;\\ \; & \;\;A_{L}\left(1\right)\;\; & \;\; & \;\\ \; & \;\; & \;\ddots \; & \;\\ \; & \;\; & \;\; & \;\;A_{L}\left(N-1\right)\; \end{array}\;\right]$$

Substituting Equations (12) and (20) into Equation (19) leads to:

(21) $$\begin{array}{cc} y & =\Theta \tilde{d}=\Theta M\bar{d}\\ & =\Theta M\left(A_{L}r_{L}+w\right)=\Upsilon r_{L}+w_{c} \end{array}$$

where $w_{c}=\Theta Hw$ is the subsampled Gaussian white noise received in the FC. Here, $\Upsilon =\Theta MA_{L}$ is the joint sparse representation matrix that combines the joint sparse matrices **Θ** and $A_{L}$. The details of this joint sparse representation matrix are:

(22) $$\Upsilon \;=\;\underset{\Upsilon [1]}{\underset{︸}{\left[\;\begin{array}{c} \phi_{e,1}^{1}a_{L,1}^{T}\left(0\right)\\ \phi_{e,1}^{2}a_{L,2}^{T}\left(0\right)\\ \vdots \\ \phi_{e,1}^{H}a_{L,H}^{T}\left(0\right) \end{array}\right.}}\underset{\Upsilon [2]}{\underset{︸}{\begin{array}{c} \phi_{e,2}^{1}a_{L,1}^{T}\left(1\right)\\ \phi_{e,2}^{2}a_{L,2}^{T}\left(1\right)\\ \vdots \\ \phi_{e,2}^{H}a_{L,H}^{T}\left(1\right) \end{array}}}\begin{array}{c} \ddots \end{array}\underset{\Upsilon [N]}{\underset{︸}{\left.\begin{array}{c} \phi_{e,N}^{1}a_{L,1}^{T}\left(N-1\right)\\ \phi_{e,N}^{2}a_{L,2}^{T}\left(N-1\right)\\ \vdots \\ \phi_{e,N}^{H}a_{L,H}^{T}\left(N-1\right) \end{array}\;\right]\;}}$$

where $\phi_{e,n}^{h}$ is the *n*-th column of $\Phi_{e}^{h}\Psi$ and $a_{L,h}\left(n\right)$ is the *h*-th row of $A_{L}\left(n\right)$. The significance of Equation (22) is that one may reconstruct the joint sparse indicating vector $r_{L}$ from the joint measurement vector y directly without reconstructing the raw data $x_{h},1\leq h\leq H$ at individual sensor nodes. $r_{L}$ is a joint sparse vector with no more than *R* non-zero blocks, and there are at most *Q* non-zero entries in each of the *R* non-zero blocks.

With these relations, we may now formulate the joint sparse representation-based DoA estimation problem (group sparse reconstruction [33]) in a *multiple measurements vector* (MMV) formulation [26,34,35]:

(23) $$\min_{r_{L}}\sum_{\ell =1}^{L}|s\left(\ell \right)|\;\text{subject}\;\text{to}\;||y-\Upsilon r_{L}{\left|\right|}_{2}\leq \gamma$$

where $s\left(\ell \right)=\sum_{n=0}^{N-1}r_{\ell }{\left(n\right)}^{2}$ and *γ* is a noise threshold. To solve this optimization problem, a second-order cone programming (SOCP) [36] approach has been proposed. The joint sparse representation of the wideband array signal in Equation (23) helps to improve the DoA estimation performance of wideband signals, as long as the same DoA of a signal is shared by different frequencies [37]. Note that the group sparsity-based DoA estimation for wideband signals is advantageous as compared to the traditional wideband DoA estimation methods, because it achieves near-coherent DoA estimation performance without the requirement of coherent signal projection through, e.g., the well-known focusing technique [38].

However, the computation cost is rather expensive. In [39], an MMV-Proxmethod has been proposed. We adopt this method for the underlying problem. In particular, we solve Equation (23) by a two-step process:

1. Estimate the frequency domain support T of array signals by reconstructing the spectral sparse indicative vector $\tilde{d}$ from the joint measurements y and prune the joint reconstruction matrix **Υ** by selecting Υ[r] with non-zero frequency bins;
2. reconstruct the DoA indicative vector s directly from the joint measurements y using the pruned joint reconstruction matrix.

In summary, the first step is to estimate the non-zero block $r_{L}\left(k\right)$ and to simplify the joint sparse matrix by selecting Υ[r] with non-zero $r_{L}\left(r\right)$. The second step is to reconstruct the DoA using the pruned joint sparse representation matrix $\tilde{\Upsilon }$. These steps are summarized in the listing of Algorithm 1.

The computational complexity of Equation (23) in the SOCP framework using an interior point implementation is $O(L^{3}N^{3})$ [39]. If we decouple the original problem into two sub-problems (Steps 2, 3 and 4 in the above algorithm), its computational complexity can be reduced to $O(L^{3}R^{3}+N^{3}H^{3})$.

**Algorithm 1** Decoupled joint sparse reconstruction.Input: Received joint random measurement vector y;Equivalent random measurement matrix $\Phi_{e}^{h}$;Output: DoA indicating vector s;1. Estimate the noise level by random sampling in source free scenario, $\gamma =\left||y\right|{|}_{2}^{2}$;2. Estimate the common support $T=\mathrm{supp}(\tilde{d})$ by solving: $\begin{array}{c} \min_{\tilde{d}}\sum_{n=0}^{N-1}\sum_{h=1}^{H}d_{h}{\left(n\right)}^{2}\\ \text{subject to }||y-\Theta \tilde{d}{\left|\right|}_{2}^{2}\leq \gamma ; \end{array}$3. Construct the pruned joint reconstruction matrix $\tilde{\Upsilon }$;$\tilde{\Upsilon }=\left[\Upsilon \left[n_{1}\right],\Upsilon \left[n_{2}\right],\cdots ,\Upsilon \left[n_{\left|T\right|}\right]\right],r\in T$;4. Solve the pruned reconstruction problem: $\begin{array}{c} \min_{{\bar{r}}_{L}}{∥s∥}_{1}\text{subject to }||y-\tilde{\Upsilon }\bar{r}{\left|\right|}_{2}^{2}\leq \gamma ,\\ s\left(\ell \right)=\sum_{n\in T}r_{\ell }{\left(n\right)}^{2},\bar{r}={\left[r^{T}\left(n_{1}\right),\cdots ,r^{T}\left(n_{\left|T\right|}\right)\right]}^{T}; \end{array}$

## 4. Performance Analysis

In this section, the performance of the proposed CSJSR-DoA approach is investigated. First, the relevant coherence, its upper bound and the angular separation problem are studied. Second, the Cram$\overset{´}{e}$r–Rao bound of the proposed CSJSR-DoA approach will be discussed.

### 4.1. Sparse Reconstruction Analysis and Angle Separation

The existence of solution to Equation (23) depends on the property of the reconstruction dictionary **Υ**. Usually, the mutual coherence and the restricted isometry property (RIP) of a sparse representation matrix are used as the criteria that indicate the reconstruction performance. Consider that the verification of the RIP property requires combinational computational complexity [40]; it is preferable to use the property of coherence that is easily computable to provide more concrete recovery guarantees. In this paper, we try to analyze the coherence of the CSJSR-DoA approach.

In the CS theory, the coherence of a matrix is the worst-case linear dependence of any pair of its column vectors. Similarly, in the block sparse signal case, in which a signal is composed by several blocks and only a small number of these blocks is non-zero, the block-coherence [41] has been proposed.

Recall from Equation (21):

(24) $$\Upsilon =\Theta MA_{L}$$

where **Θ** is a block diagonal joint sparse sampling matrix, M is a full rank, unitary permutation matrix and $A_{L}$ is a block diagonal steering matrix. To study the coherence of **Υ**, we need to study the block-coherence of **Υ**.

**Theorem 1.**  *Assume that H sensor nodes form a sensor array, and the received data in the fusion center are $y_{h}=\Phi_{e}^{h}x_{h}\left(\Phi_{e}^{h}\in R^{M\times N}\right),h=1,2,\cdots ,H$. Then, the block-coherence of the joint sparse representation matrix* **Υ** *satisfies:*

(25) $$\mu_{B}\left(\Upsilon \right)\leq \sqrt{\frac{N-M}{(N-1)M}}$$

**Proof.**  See Appendix A. ☐

The block-coherence $\mu_{B}$ guarantees stable reconstruction of the block sparse signal $r_{L}$ with a high probability. However, each block $r_{L}\left(n\right)$ indicates the DoA of sources. Thus, the coherence of each sub-matrix Υ[n] should be considered. In this paper, we derive the coherence of Υ[n]. This result will provide a lower bound of the source separation (in turns of DoA angle) issue to ensure a reliable DoA estimation. However, the coherence of Υ[n] depends on array geometry, and it is difficult to obtain an analytical bound.

In this paper, we consider the typical uniform linear array geometry and find an interesting result of the coherence bound. The derived result is similar to the array pattern [42] analysis and is validated for both narrowband and broadband acoustic sources. For broadband sources, the highest dominant frequency may be used.

**Theorem 2.**  *Assume that H sensor nodes form a uniformly-spaced linear array with d(≤λ/2) being the spacing between adjacent sensors where λ is the wavelength of the acoustic signal. If the difference of the incident angles of any pair of sources satisfies:*

(26) $$\Delta \theta \geq \frac{c}{Hdf}$$

*then the coherence of Υ[n] is bounded by:*

(27) $$\mu \left(\Upsilon \left[n\right]\right)\leq \frac{1}{H\sin(\pi /H)}\sqrt{\frac{N-M}{(N-1)M}}$$

**Proof.**  See Appendix B. ☐

### 4.2. Cram$\overset{´}{e}$r–Rao Bound of CSJSR-DoA

In this subsection, we derive the Cram$\overset{´}{e}$r–Rao bound (CRB) of the proposed CSJSR-DoA method and study its impact on the DoA estimation accuracy due to factors such as the number of measurements, the number of sensors and the noise level $\sigma^{2}$, which is defined in Equation (13).

Recall that the CRB is defined as the inverse of the Fisher information matrix (FIM) [43],

(28) $$\mathrm{CRB}\left(\Lambda \right)=F^{-1}\left(\Lambda \right)$$

where **Λ** is the unknown parameter set. In this paper, we only derive the CRB of a single source case. Thus, the unknown parameter is $\Lambda ={\left[\theta ,r^{T},f^{T}\right]}^{T}$, where $f={\left[f_{1},f_{2},\cdots ,f_{R}\right]}^{T}$ and $r={\left[r_{0}\left(k_{1}\right),r_{0}\left(k_{2}\right),\cdots ,r_{0}\left(k_{R}\right)\right]}^{T}$ are the sparse frequency bins and their corresponding source spectrum, respectively.

In the case of Gaussian white noise, the FIM is:

(29) F(Λ)=σ22Re[GHG]

where $G=\partial y/\partial \Lambda^{T}$. For simplification, we assume that all *H* sensors have the same $\Phi_{e}\left(\Phi_{e}^{1}=,\cdots ,=\Phi_{e}^{H}=\Phi_{e}\right)$. Recall Equations (4), (6) and (17); the measurement vector of the CSJSR-DoA approach in a single source case is:

(30) $$y\left(\Lambda \right)=\sum_{i=1}^{R}r_{0}\left(k_{i}\right)a_{0}\left(k_{i}\right)⊗\phi_{e,k_{i}}+w_{c}$$

where $\phi_{e,k_{i}}$ is the $k_{i}$-th column of $\Phi_{e}\Psi$. After some calculations, the CRB of the CSJSR-DoA approach is given by:

(31) $$CRB\left(\theta \right)=\frac{\sigma^{2}}{{2M\alpha ∥e∥}_{2}^{2}}$$

where $e={\left[f_{1}r_{0}\left(k_{1}\right),f_{2}r_{0}\left(k_{2}\right),\cdots ,f_{R}r_{0}\left(k_{R}\right)\right]}^{T}$, $\alpha =\frac{4\pi^{2}}{c^{2}}\sum_{h=0}^{H-1}{\left(u_{h}\cos\left(\theta \right)-v_{h}\sin\left(\theta \right)\right)}^{2}$.

**Proof.**  See Appendix C. ☐

## 5. Performance Evaluation

In this section, extensive simulations are carried out to: (1) study the performance of the CSJSR-DoA approach; and (2) compare the performance of the CSJSR-DoA approach against COBE and CSA-DoA, using the L1-SVD [26] algorithm as the baseline. In addition, we also developed a hardware prototype platform and collected data from outdoor experiments to validate the practical applicability of the CSJSR-DoA approach.

### 5.1. Simulation Settings

We synthesized acoustic signals based on Equation (1). The synthesized signals contain four dominant frequency entries at 300 Hz, 500 Hz, 600 Hz and 800 Hz. These correspond to wavelengths of 1.14, 0.7, 0.57 and 0.43 m, respectively, assuming the sound speed at 343 m/s. The received acoustic signal also contains additive Gaussian white noise with zero mean. The variances are set so that the resulting SNR ranges from -10 dB to 10 dB in 5-dB increments. Such an acoustic signal resembles the dominant entries’ distribution of some practical acoustic sources, such as truck engine sounds. The sampling rate is 2 ksamples/s. At this rate, a frame of 125 ms is selected. The sampling rate is chosen so that it satisfies the Nyquist sampling rate and avoids the frequency aliasing issue.

We assume a single uniform linear array consisting of six (H=6) acoustic sensor nodes deployed in a sensor field. The spacing between adjacent sensors is 0.2 m, which is smaller than half of the wavelength (0.43 m) of the 800-Hz entry. All acoustic sources are located at the broad side of this linear array, so that the incident angles (directions of arrivals) are constrained within a $-90^{\circ }$ to $90^{\circ }$ range. We divide this angle range into (L=360) partitions, so that each partition equals $0.5^{\circ }$.

Consider that data packets transmitted from the sensor array to the FC may suffer from data packet loss. We will simulate data packet loss rates, denoted by $r_{\mathrm{loss}}$, at 0, 5%, 10%, 20% and 30%, respectively. To mitigate burst data transmission loss, the acoustic data stream will be interleaved while being assembled into the data packet for transmission. However, other than packet headers, there will be no additional channel coding bits appended. The data loss rate is computed as the percentage of packets that are lost during transmission through wireless channels *versus* the total number of packets that are sent from the sensor array.

With the CSJSR-DoA approach, both random subsampling and wireless channel packet loss will reduce the amount of received acoustic samples compared to what was originally sampled at the sensor array. In particular, at each sensor node, the acoustic data samples will be randomly subsampled according to Equations (14) and (15). The ratio M/N in these equations can be regarded, in the context of CS, as the ratio of the number of measurements *versus* the total number of data samples. With channel data packet loss taken into account, we instead define $r_{\mathrm{dc}}\left(\leq 1\right)$ to be the ratio of the number of acoustic samples successfully received at the fusion center *versus* the total number of samples acquired by the *H* acoustic sensors. Ignoring the overhead of data packet header, we have:

(32) $$r_{\mathrm{dc}}\approx \left(1-r_{\mathrm{loss}}\right)\times M/N$$

In this section, the DoA performance will be reported against different values of $r_{\mathrm{dc}}$. In practice, if $r_{\mathrm{loss}}$ can be estimated in real time, we may adjust *M* to achieve the desired DoA accuracy.

Four algorithms are implemented: L1-SVD, CSJSR-DoA, CSA-DoA and COBE. The L1-SVD algorithm [26] is applied to the entire set of acoustic samples without random subsampling and is used as a baseline for both simulation and experiment. The COBE algorithm [13] requires a specific reference node to be sampled at the Nyquist rate without any subsampling in order to provide a reference source signal. To have fair comparisons, we performed trial runs to empirically obtain the best parameters for the CSA-DoA and COBE algorithms. All four algorithms are implemented using MATLAB Version 7.9.0. The optimization is performed using the Sedumi 1.2.1 toolbox [44].

### 5.2. CSJSR-DoA Performance Evaluation

In this simulation, two (Q=2) stationary acoustic sources are placed in the far field from the sensor array at $\left[-60^{\circ },30^{\circ }\right]$. The noise levels of the acoustic signal correspond to SNR = -10 dB, -5 dB, 0 dB, 5 dB and 10 dB, and it is assumed that $r_{\mathrm{loss}}=0\%$. Five hundred independent trials are performed. Consider that sparse reconstruction is a probability event; a DoA estimate is considered a *success detection* if a sparse indicative vector can be reconstructed successfully. In other words, some trials fail to give a solution to the sparse DoA indicative vector. Based on the results of detected trials, the number of impinging signals, *Q*, and the corresponding DoAs can be estimated. Similar to some spatial spectrum search-based approaches, the spatial spectrum will be calculated using $r_{\mathrm{ss}}=20\log_{10}\left(s/\max(s)\right)$, and some conventional peak-finding approaches [45,46] are used to find $\tilde{Q}$ peaks of the obtained spatial spectrum. However, the estimated $\tilde{Q}$ directions can be divided into three categories: $\tilde{Q}<Q$, $\tilde{Q}=Q$ and $\tilde{Q}>Q$. In this case, a proper performance criterion is hard to choose. Following a similar work [22], which takes into account both the errors in estimating the signal number $\tilde{Q}$ and the corresponding DoAs, the root mean square errors (RMSE) of the *n*-th detected trial will also be reported using the formula:

(33) $$\mathrm{RMSE}=\sqrt{\frac{\sum_{n=1}^{N_{e}}{\left({\mathrm{RMSE}}^{\left(n\right)}\right)}^{2}}{N_{e}}}$$

(34) $$\mathrm{RMS}E^{\left(n\right)}=\left\{\begin{array}{c} \sqrt{\frac{\sum_{q=1}^{\tilde{Q}\left(n\right)}{\left|\theta_{q}-{\tilde{\theta }}_{q}^{\left(n\right)}\right|}^{2}+\left|Q-{\tilde{Q}}^{\left(n\right)}\right|{\left(\Delta \theta_{\max}\right)}^{2}}{Q}},{\tilde{Q}}^{\left(n\right)}\leq Q\\ \sqrt{\frac{\sum_{q=1}^{Q}{\left|\theta_{q}-{\tilde{\theta }}_{q}^{\left(n\right)}\right|}^{2}+\sum_{j=Q+1}^{{\tilde{Q}}^{\left(n\right)}}{\left|{\tilde{\theta }}_{j}^{\left(n\right)}-{\bar{\theta }}_{j}^{\left(n\right)}\right|}^{2}}{Q}},{\tilde{Q}}^{\left(n\right)}>Q \end{array}\right.$$

where $N_{e}$ is the number of detected trials, ${\bar{\theta }}_{j}^{\left(n\right)}=\arg\left\{\min_{\theta_{q},q\in \left[1,Q\right]}\left|\theta_{q}-{\tilde{\theta }}_{j}^{\left(n\right)}\right|\right\}$. Here, $\Delta \theta_{\max}$ is a penalty term of the maximum admissible DoA error (*i.e*., $180^{\circ }$ for a liner array), and ${\bar{\theta }}_{j}^{\left(n\right)}$ is the least error of additional false DoA.

**Figure 4:**
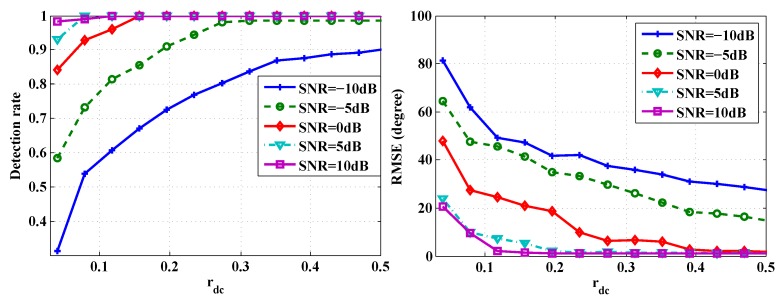
Performance comparison of the CSJSR-DoA approach under different data volumes: (**left**) detection rate (**right**) RMSE.

The averaged detection rate and the RMSE are summarized in Figure 4. It may be noted that when the SNR is greater or equal to 0 dB, $r_{\mathrm{dc}}\geq 0.25$ yields very satisfactory DoA performance. To put it into context, $r_{\mathrm{dc}}=0.25$ roughly equals transmitting 500 samples per second without data loss. The corresponding data volume of Nyquist rate sampling is 1600.

**Figure 5:**
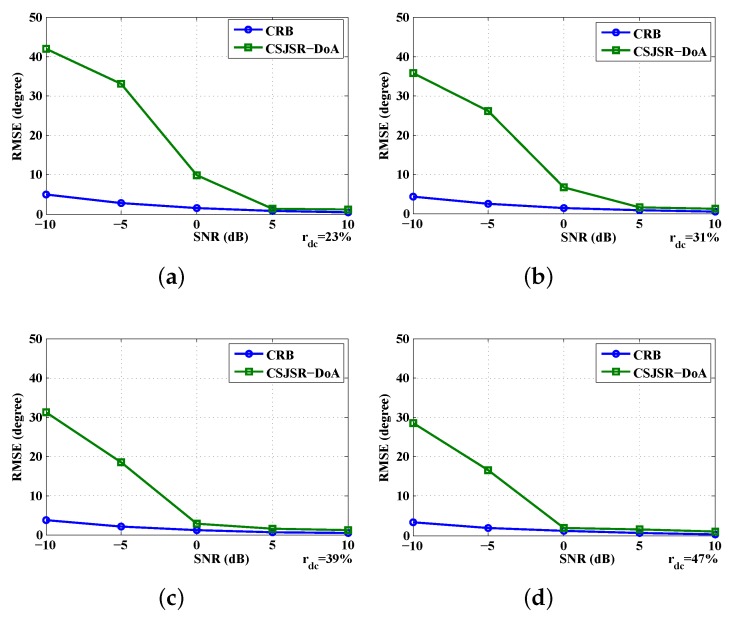
Comparison between the CSJSR-DoA result and the Cram$\overset{´}{e}$r–Rao bound (CRB): (**a**) CRB comparison with $r_{dc}=23\%$; (**b**) CRB comparison with $r_{dc}=31\%$; (**c**) CRB comparison with $r_{dc}=39\%$; (**d**) CRB comparison with $r_{dc}=47\%$.

It is also interesting to examine how the RMSE obtained using simulation is compared against the theoretical Cram$\overset{´}{e}$r–Rao bound. In Figure 5, it can be seen that for SNR>0 dB, the RMSE is very close to the theoretically-computed CRB.

**Figure 6:**
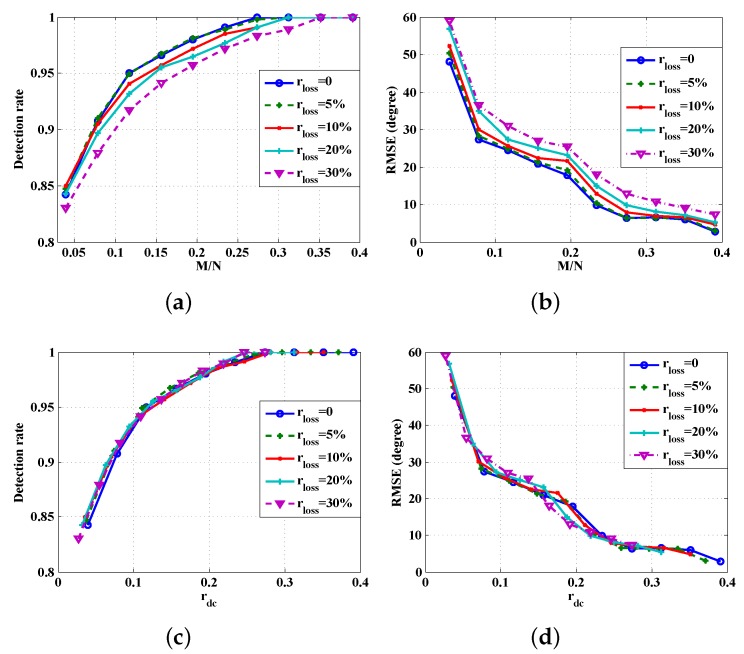
Comparison of different lossy transmissions: (**a**) Detection rate *versus* M/N; (**b**) RMSE *versus* M/N; (**c**) Detection rate *versus* $r_{dc}$; (**d**) RMSE *versus* $r_{dc}$ .

Next, we want to investigate the impact of varying the data packet loss rate for different values of random subsampling ratio M/N. The noise level of the acoustic signal is set at SNR = 0 dB. Two sets of plots are provided in Figure 6. On the top, the averaged probability of successful detection and the RMSE are plotted against M/N. The performance degradation due to data packet loss can be seen clearly. On the bottom, the averaged probability of successful detection and the RMSE are plotted against $r_{\mathrm{dc}}$, which, according to Equation (32), already takes into account the impact of $r_{\mathrm{loss}}$. Hence, the different curves on the bottom panel are merged into an identical one. This is because the random data loss is equivalent to the random subsampling of the raw data.

Our next goal is to investigate the DoA resolution of the proposed CSJSR-DoA approach when two acoustic sources become closer in terms of DoAs. Under the same simulation configuration, we fix one source at $0^{\circ }$ and vary the incident angle of the second source from $2^{\circ }$ to $40^{\circ }$ in $2^{\circ }$ increments. We perform 300 independent trials for each setting. The SNR=5dB, and the peaks of the 360×1 sparse vector indicate the DoAs of two sources.

**Figure 7:**
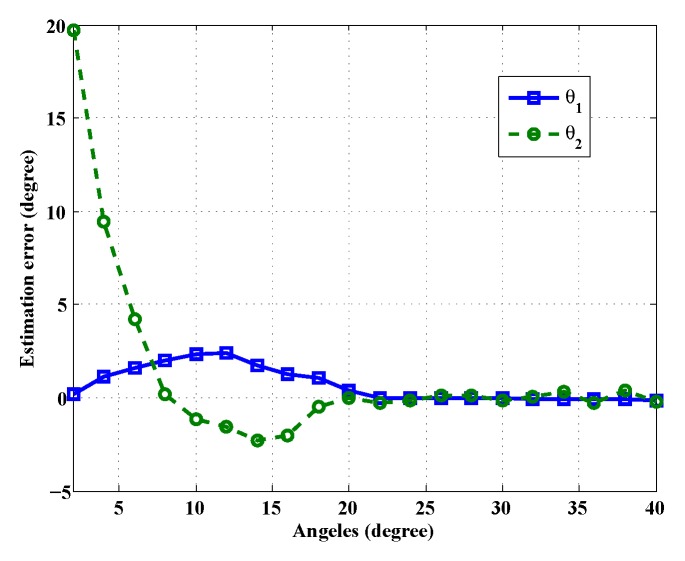
Comparison of DoA estimation error under different angle separations.

**Figure 8:**
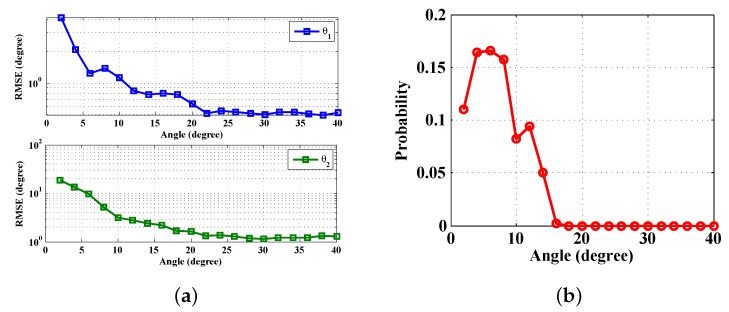
Angle separation comparison: (**a**) RMSE *versus* angle (**b**) false probability *versus* angle.

We report the averaged angle estimation error *versus* target DoA separation angles in Figure 7. Based on the discussion on Theorem 2, the steering vectors of two nearby sources are strongly correlated or have larger coherence. Therefore, the reconstruction of a correct sparse DoA indicative vector does not perform well. In simulations, the probability (false DoA probability) that only one source is found when two DoAs exist and the RMSE of each DoA estimation *versus* target DoA separation angles are shown in Figure 8. Note that when the separation angles are small, the averaged estimation errors, the RMSEs and the probability of false DoA estimation are rather large. However, when the targets are well separated ($\geq 20^{\circ }$), the DoAs of both targets are accurately estimated (the averaged estimation error and the false DoA probability are close to zero, and the RMSEs decrease to a constant). This validates the theoretical angle separation result of $\frac{c}{Hdf}=\frac{343}{6\times 0.2\times 800}=20.5^{\circ }$.

**Figure 9:**
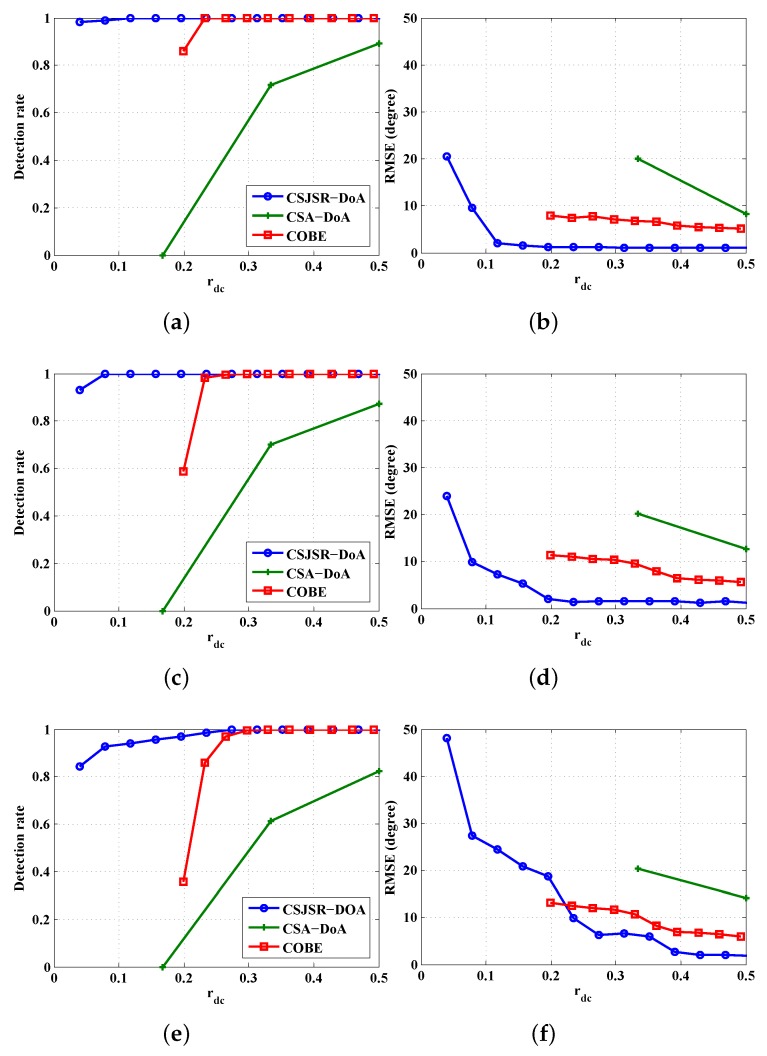
Performance comparison among CS-based methods under the same SNR ratio:(**a**) Detection rate with SNR = 10 dB; (**b**) RMSE with SNR = 10 dB; (**c**) Detection rate with SNR = 5 dB;(**d**) RMSE with SNR = 5 dB; (**e**) Detection rate with SNR = 0 dB; (**f**) RMSE with SNR = 0 dB.

Finally, we use simulation to compare the proposed CSJSR-DoA approach against two other CS-based DoA methods: COBE and CSA-DoA. The simulation conditions are identical to those used in Figure 4. While these three methods differ in how the measurements are made, the definition of $r_{\mathrm{dc}}$ would still be applicable to all of them; namely, the number of samples received at the FC *versus* the total amount of raw data samples at the sensor array. Specifically, the analog projection of the CSA-DoA approach is emulated by projecting the digitized signal. The results are summarized in Figure 9. It shows that the CSJSR-DoA method has favorable performance over others in a number of situations. Additional comparison of these methods using data collected from a prototype WSAN platform will be discussed next.

### 5.3. Prototype WSAN Platform and Field Experiment

**Figure 10:**
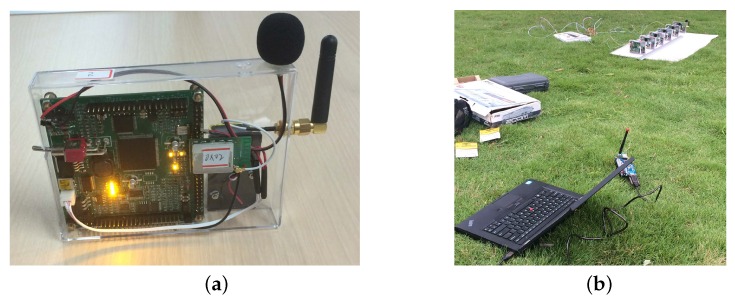
Wireless sensor array network. (**a**) Sensor node; (**b**) fusion center and array.

To further validate the capability of the CSJSR-DoA approach, a WSAN [47] was developed. Each sensor node (shown in Figure 10a) is equipped with an omni-directional microphone, a 16-bit ADC and a wireless transceiver operating at a rate of 31.25 KBps using the ZigBee protocol [48]. Random sub-sampled acoustic data samples are transmitted to the FC through the wireless transceiver to the FC (laptop). To guarantee time synchronization within the sensor array, the RBS time synchronization scheme is used. Figure 10b shows the experiment configuration, in which a laptop equipped with a wireless receiver was used as the FC. The sensor array is shown in the background close to the upper right corner.

**Figure 11:**
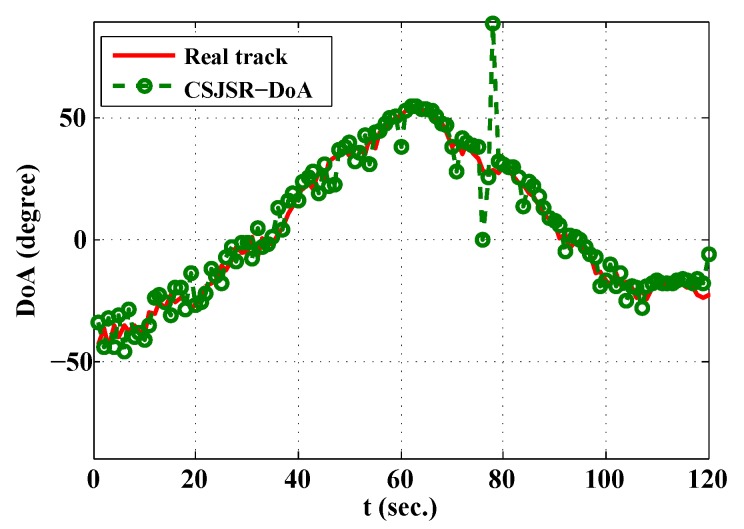
Experiment result of the CSJSR-DoA approach.

In the first experiment, the task is to track the DoAs of a single moving acoustic source (digital acoustic signal played through a speaker). The distance between the source and the array varies from 20 m to 40 m, and the measured SNR in the array was about 6 dB. Each sensor node took samples during a period of 125 ms at a 2 Ksampling rate and transmitted them to the FC using the IEEE 802.15.4 protocol. In the FC, the DoA estimate was updated every second. The measured data loss ratio $r_{\mathrm{loss}}$ was 0.27, and the corresponding $r_{\mathrm{dc}}$ of the received data in the FC was 0.23. The result is shown in Figure 11. The red line is the ground truth, and the green circles are CSJSR-DoA estimates. The averaged probability of success detection is 90%, and the corresponding RMSE is $2.76^{\circ }$. This experiment provides an example of the practical use of the CSJSR-DoA approach.

**Figure 12:**
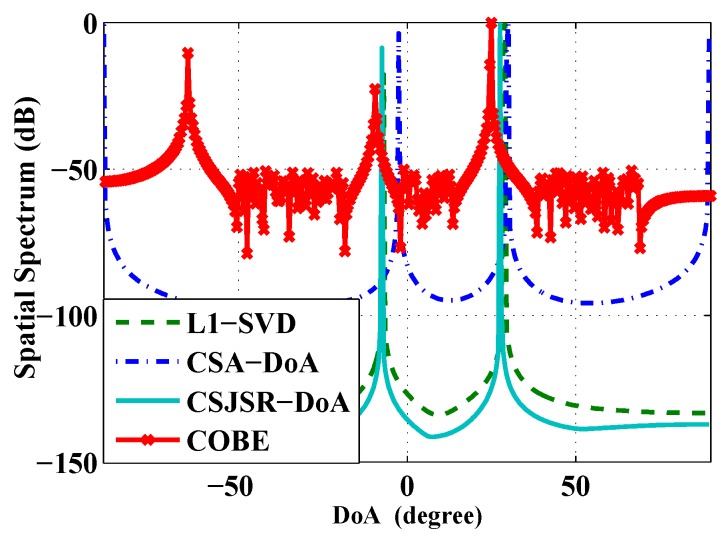
Prototype system experiment of the CSJSR-DoA approach.

In the second experiment, two stationary acoustic sources are used. They are placed at distances of 12.94 m and 14.66 m with (ground truth) DoA angles at $-6.5^{\circ }$ and $31.0^{\circ }$ respectively. Six sensors were incorporated into the array with an inter-sensor spacing equal to 0.2 m. Using data collected in this experiment, the performance of the CSJSR-DoA approach is compared against COBE, CSA-DoA and the baseline algorithm L1-SVD. The results are shown in Figure 12 and Table 2. The data compression ratio of all of these algorithms is fixed at $r_{\mathrm{dc}}=0.3$. To facilitate the comparison, the corresponding L×1 sparse DoA angle vector s is normalized and expressed in units of dB. From Figure 12, it is shown that the CSJSR-DoA approach yields sharper DoA estimates compared to the other three methods.

## 6. Conclusions

In this paper, a compressive sensing joint sparse representation approach (CSJSR-DoA) is presented for DoA estimation on a WSAN platform. By exploiting both frequency domain and spatial domain sparsity, the CSJSR-DoA approach provides a direct DoA angle estimation at the FC, while requiring almost no computation at power-constrained remote sensor nodes. We provided performance analysis in terms of DoA angle resolution and the Cram$\overset{´}{e}$r–Rao bound of the estimates. We further conducted extensive simulation and built a prototype experimental WSAN platform to investigate the impacts of various parameters on the DoA performance. We also compared the performance of the proposed CSJSR-DoA approach against two other compressive sensing DoA estimation methods and showed that the CSJSR-DoA approach provides superior performance in both simulation runs and real-world experiments.

## Figures and Tables

**Table 1:** Symbols and notations. CSJSR, compressive sensing joint sparse representation.

Symbol	Explanation
*H*	number of sensors within an array
*Q*	number of sources
$R_{q}$	sparsity of the *q*-th source signal
$s_{q}$	the *q*-th source signal in the time domain
$r_{q}$	dominant sparse vector in the frequency domain with $R_{q}$-sparsity
$\nu_{q}$	less prominent components of $s_{q}$ in the frequency domain
$\tau_{h,q}$	time delay of the *q*-th source between the *h*-th sensor and the array centroid
$x_{h}\left(t\right)$	received time domain signal in the *h*-th array
**Ψ**	N×N inverse DFT matrix
$x_{h}$	received time domain signal at the *h*-th sensor, $x_{h}={\left[x_{h}\left(1\right),\cdots ,x_{h}\left(N\right)\right]}^{T}$
${\tilde{d}}_{h}$	signal spectrum of $x_{h}$, ${\tilde{d}}_{h}={\left[d_{h}\left(0\right),d_{h}\left(1\right),\cdots ,d_{h}\left(N-1\right)\right]}^{T}$
d(k)	array data spectrum at the *k*-th frequency, $d\left(k\right)={\left[d_{1}\left(k\right),d_{2}\left(k\right),\cdots ,d_{H}\left(k\right)\right]}^{T}$
$a_{\ell }\left(k\right)$	steering vector of $\theta_{\ell }$ at the *k*-th frequency,
	$a_{\ell }\left(k\right)={\left[\exp\left(-j\omega_{k}\tau_{1,q}\right),\cdots ,\exp\left(-j\omega_{k}\tau_{H,q}\right)\right]}^{T}$
r(k)	source signal vector of *L* directions, $r\left(k\right)={\left[r_{1}\left(k\right),r_{2}\left(k\right),\cdots ,r_{L}\left(k\right)\right]}^{T}$
$A_{L}\left(k\right)$	steering matrix of *L* directions at the *k*-th frequency
	$A_{L}\left(k\right)=\left[a_{1}\left(k\right),a_{2}\left(k\right),\cdots ,a_{L}\left(k\right)\right]$
D	array data spectrum matrix
M	permutation matrix
$\bar{d}$	wideband array data spectrum, $\bar{d}={\left[d^{T}\left(0\right),d^{T}\left(1\right),\cdots ,d^{T}\left(N-1\right)\right]}^{T}$
$\tilde{d}$	array spectrum of *H* sensors, $\tilde{d}={\left[{\tilde{d}}_{1}^{T},{\tilde{d}}_{2}^{T},\cdots ,{\tilde{d}}_{H}^{T}\right]}^{T}$
u	sample intervals, $u={\left[u\left(1\right),u\left(2\right),\cdots ,u\left(M\right)\right]}^{T}$
r(·)	rounding operation
**Φ**	random sub-sampling matrix
$\Phi_{loss}^{h}$	channel loss matrix
$y_{h}$	received measurement of the *h*-th sensor in the fusion center
y	joint measurement vector of *H* sensors, $y={\left[y_{1}^{T},\cdots ,y_{H}^{T}\right]}^{T}$
w	joint noise vector of *N* frequencies, $w={\left[w(0),w(1),\cdots ,w(N-1)\right]}^{T}$
**Θ**	joint measurement matrix of *H* sensors, $\Theta =\mathrm{diag}(\Phi_{e}^{1}\Psi ,\Phi_{e}^{2}\Psi ,\cdots ,\Phi_{e}^{H}\Psi )$
diag(.)	block diagonal matrix operation
**Υ**	joint sparse matrix
s	direction indicative vector, $s\left(\ell \right)=\sum_{n=0}^{N-1}r_{\ell }{\left(n\right)}^{2}$
supp(.)	nonzero index of a vector, $\mathrm{supp}\left(x\right)=\left\{n|x(n)>0\right\}$
$\tilde{\Upsilon }$	pruned joint sparse matrix, $\tilde{\Upsilon }=\left[\Upsilon \left[n_{1}\right],\Upsilon \left[n_{2}\right],\cdots ,\Upsilon \left[n_{\left|T\right|}\right]\right],r\in T$
F(Λ)	Fisher information matrix of parameter **Λ**
CRB	Cram$\overset{´}{e}$r–Rao bound of the CSJSR algorithm

**Table 2:** Result of the prototype system experiment. CSA, compressive sensing array; COBE, compressive bearing estimation.

	L1-SVD	CSJSR-DoA	CSA-DoA	COBE
DoA (${}^{\circ }$)	[-7,29]	[-7.5,27.5]	[-2.5,30]	[-9.5,25]

**Figure B1:**
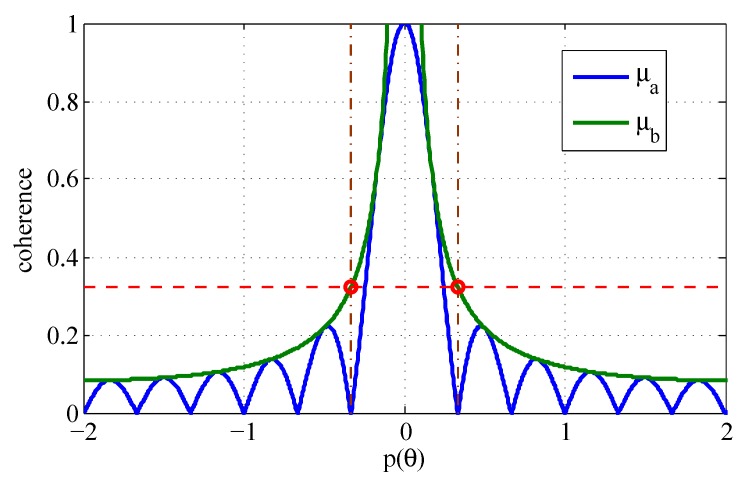
Mutual coherence of the array manifold matrix and its upper bound.

## Appendix A.

Let $C=[\underset{C[1]}{\underset{︸}{c_{1}\cdots c_{d}}},\underset{C[2]}{\underset{︸}{c_{d+1}\cdots c_{2d}}},\cdots \underset{C[M]}{\underset{︸}{c_{N-d+1}\cdots c_{N}}}]$ be a concatenation of column block C[m] of size L×d. Then, the block coherence is defined as:

(A1) $$\begin{array}{c} \mu_{B}=\max_{r,l\neq r}\frac{1}{d}\rho \left(C{\left[r\right]}^{H}C\left[l\right]\right) \end{array}$$

where ρ(X) is the spectral norm of matrix X. For simplification, we assume all of the *H* sensors have the same measurement matrix ($\Phi_{e}=\Phi_{e}^{1}=,\cdots ,=\Phi_{e}^{H}$); therefore:

(A2) $$\begin{array}{c} \begin{array}{cc} \Upsilon {\left[l\right]}^{H}\Upsilon \left[r\right] & =\frac{1}{MH}{\left(A_{L}\left(l\right)⊗\phi_{e,l}\right)}^{H}\left(A_{L}\left(r\right)⊗\phi_{e,r}\right)\\ & =\frac{\phi_{e,l}^{H}\phi_{e,r}}{MH}A_{L}{\left(l\right)}^{H}A_{L}\left(r\right) \end{array} \end{array}$$

Note the structure of $\phi_{e,r}$; the product $\Phi_{e}\Psi$ is equivalent to selecting some rows from the discrete Fourier transform (DFT) matrix **Ψ**. Hence, their product $\Phi_{e}\Psi$ is a partial Fourier transform matrix (submatrix of a full DFT matrix). For the underlying random non-uniform sampling, the selection of these *M* rows is determined by u. Applying the Welch Bound inequality [49], when u is randomly selected from {1,2,⋯,N},

(A3) $$\begin{array}{c} |\phi_{e,l}^{H}\phi_{e,r}|\leq \sqrt{\frac{(N-M)M}{\left(N-1\right)}} \end{array}$$

According to the Gershgorin circle theorem, $\mu_{B}$ is bound by:

(A4) $$\begin{array}{c} \begin{array}{cc} \mu_{B} & =\max_{l,r\neq l}\frac{1}{L}\rho \left(\Upsilon {\left[l\right]}^{H}\Upsilon \left[r\right]\right)\\ & \leq \max_{l,r\neq l}\frac{1}{LH}\sqrt{\frac{\left(N-M\right)}{(N-1)M}}\lambda_{\max}\left(A_{L}{\left(l\right)}^{H}A_{L}\left(r\right)\right)\\ & =\sqrt{\frac{N-M}{(N-1)M}} \end{array} \end{array}$$

## Appendix B.

Assume that a uniform linear array consists of *H* sensors and that the distance between adjacent sensors is *d*. The orientationof a linear array is set to be orthogonal to the y-axis, and the array centroid is [0,0]. The positions of the *h*-th sensor satisfies $u_{h}-u_{h-1}=d\left(h=2,3,\cdots ,H\right)$, $v_{h}=0\left(h=1,2,\cdots ,H\right)$ and $\tau_{h,q}=u_{h}\sin\left(\theta_{q}\right),\theta_{q}\in \left(-90,90\right]$. In this case, the corresponding steering vector is given by:

(B1) $$\begin{array}{c} \begin{array}{cc} a_{\ell }\left(k\right) & =\;{\left[e^{\frac{-j\omega_{k}u_{1}\sin\left(\theta_{\ell }\right)}{c}},e^{\frac{-j\omega_{k}u_{2}\sin\left(\theta_{\ell }\right)}{c}},\cdots ,e^{\frac{-ju_{H}\omega_{k}\sin\left(\theta_{\ell }\right)}{c}}\right]}^{T}\\ & =\chi {\left[1,e^{\frac{-j\omega_{k}d\sin\left(\theta_{\ell }\right)}{c}},\cdots ,e^{\frac{-ju_{H}\omega_{k}\left(H-1\right)d\sin\left(\theta_{\ell }\right)}{c}}\right]}^{T} \end{array} \end{array}$$

where $\chi =e^{\frac{-j\omega_{k}u_{1}\sin\left(\theta_{\ell }\right)}{c}}$ is a constant. Recall Equation (A2); the block sub-matrix is given by:

(B2) $$\begin{array}{c} \Upsilon \left[k\right]=A_{L}\left(k\right)⊗\phi_{e,k} \end{array}$$

The sparse representation matrix in Equation (8) that compactly expresses the spectrum of the *H* received signals is:

(B3) $$\begin{array}{c} A_{L}\left(k\right)=\left[a_{1}\left(k\right),a_{2}\left(k\right),\cdots ,a_{L}\left(k\right)\right] \end{array}$$

when $\omega_{k}d/c>\pi$ (d>λ/2) [37,50]; there are at least two different angles $\theta_{1}$ and $\theta_{2}$ that satisfy:

(B4) $$\begin{array}{c} e^{\frac{-j\omega_{k}d\sin\left(\theta_{1}\right)}{c}}=e^{\frac{-j\omega_{k}d\sin\left(\theta_{2}\right)}{c}} \end{array}$$

in which $\theta_{1}\neq \theta_{2}\in \left(-\pi /2,\pi /2\right]$. This implies that there will be multiple identical columns in $A_{L}\left(k\right)$, and hence, the coherence of $A_{L}\left(k\right)$ will be one. However, in CS theory, smaller coherence means better reconstruction performance. To confirm the uniqueness of $A_{L}\left(k\right)$ (not the same columns in $A_{L}\left(k\right)$) and, hence, a stable reconstruction of a sparse indicating vector, a small coherence is desirable.

On the condition that d≤1/2λ, the coherence of Υ[k] is given by:

(B5) $$\begin{array}{c} \begin{array}{c} \mu \left(\Upsilon [k]\right)\\ =\frac{1}{MH}\max_{1\leq \ell_{1}\neq \ell_{2}\leq L}\left|{\left(a_{\ell_{1}}\left(k\right)⊗\phi_{e,k}\right)}^{H}\left(a_{\ell_{2}}\left(k\right)⊗\phi_{e,k}\right)\right|\\ \leq \frac{1}{H}\sqrt{\frac{N-M}{M(N-1)}}\max_{1\leq \ell_{1}\neq \ell_{2}\leq L}\left|a_{\ell_{1}}^{H}\left(k\right)a_{\ell_{2}}\left(k\right)\right|\\ =\frac{1}{H}\sqrt{\frac{N-M}{M(N-1)}}\max_{1\leq \ell_{1}\neq \ell_{2}\leq L}\left|\sum_{h=0}^{H-1}\exp(\frac{-jh\omega_{k}dp\left(\theta \right)}{c})\right|\\ =\sqrt{\frac{N-M}{M(N-1)}}\mu_{a} \end{array} \end{array}$$

where $\mu_{a}=\frac{1}{H}\max_{1\leq \ell_{1}\neq \ell_{2}\leq L}\left|\frac{1-\exp\left(\frac{-jH\omega_{k}dp\left(\theta \right)}{c}\right)}{1-\exp\left(\frac{-j\omega_{k}dp\left(\theta \right)}{c}\right)}\right|$ is determined by the redundant array manifold matrix, $p\left(\theta \right)=\sin\left(\theta_{1}\right)-\sin\left(\theta_{2}\right)\approx \cos\left(\theta_{1}\right)\Delta \theta$, $\Delta \theta =\theta_{1}-\theta_{2}$.

Note that $\lim_{\Delta \theta \to 0}\mu_{a}=1$, that is when the incident angles of two sources are close enough, the coherence may approach $\mu_{B}$, and hence, their DoAs become unresolvable. If the angle difference between two sources is larger than a certain value, a small coherence value can be guaranteed. Based on such an observation, an upper bound of $\mu_{a}$ can be established as $\mu_{b}=2/(H|1-\exp(\frac{-j\omega d\;p(\theta_{1})}{c})\left|\right)$. In Figure B1, the coherence $\mu_{a}$ and its upper bound $\mu_{b}$ are plotted against $p(\theta_{1})$. It is observed that $\mu_{b}$ decreases sharply when $p(\theta_{1})$ is smaller than the first side lobe ($H\omega d\;p(\theta_{1})/c=2\pi$). It is not difficult to verify that when:

(B6) $$\begin{array}{c} \left|\Delta \theta \right|\geq \frac{c}{Hfd\cos(\theta_{1})}\geq \frac{c}{Hfd} \end{array}$$

$\mu_{a}\leq \mu_{b}\left(\Delta \theta \right)=\frac{1}{H}\left|\frac{2}{1-e^{\left(-j2\pi /H\right)}}\right|=\frac{1}{H\;\sin(\pi /H)}$. This means a small coherence can be guaranteed when the angle difference of the two sources is larger than a certain threshold.

## Appendix C.

Recall from Equation (29) that the details of the FIM matrix are:

(C1) F(Λ)=2σ2Re(∂yH∂θ∂y∂θ)(∂yH∂θ∂y∂rT)(∂yH∂θ∂y∂fT)(∂yH∂r∂y∂θ)(∂yH∂r∂y∂rT)(∂yH∂r∂y∂fT)(∂yH∂f∂y∂θ)(∂yH∂f∂y∂rT)(∂yH∂f∂y∂fT)

Additionally, each term of the FIM is:

(C2) $$\begin{array}{c} \begin{array}{cc} \frac{\partial y}{\partial \theta } & =\sum_{i=1}^{R}r_{0}\left(k_{i}\right)\left(b\left(k_{i}\right)⊙a_{0}\left(k_{i}\right)\right)⊗\phi_{e,k_{i}},\\ \frac{\partial y}{\partial r_{0}^{T}} & =[a_{0}\left(k_{1}\right)⊗\phi_{e,k_{1}},a_{0}\left(k_{2}\right)⊗\phi_{e,k_{2}},\cdots ,a_{0}\left(k_{R}\right)⊗\phi_{e,k_{R}}]\\ \frac{\partial y}{\partial f^{T}} & =[\frac{\partial y}{\partial f_{1}},\frac{\partial y}{\partial f_{2}},\cdots ,\frac{\partial y}{\partial f_{R}}], \end{array} \end{array}$$

where $b\left(k_{i}\right)\;\;=\;\;-j2\pi f_{k_{i}}\left[u_{1}\cos\left(\theta \right)-v_{1}\sin\left(\theta \right),u_{2}\cos\left(\theta \right)-v_{2}\sin\left(\theta \right),\cdots ,u_{H}\cos\left(\theta \right)-v_{H}\sin\left(\theta \right)\right]$, $\frac{\partial y}{\partial f_{i}}=r_{0}\left(k_{i}\right)a_{0}\left(k_{i}\right)⊗\left(v_{k_{i}}⊙\phi_{e,k_{i}}\right)$, $v_{k_{i}}\;=-j2\pi (u-1)/N$. We assume the coherence of $\Phi_{h}\Psi$ is very small and can be neglected (${\left(\phi_{e,k_{l}}\right)}^{H}\phi_{e,k_{n}}\approx 0,l\neq n$). Based on this approximation, each component of the FIM is given by:

(C3) $$\begin{array}{c} \begin{array}{cc} \frac{\partial y^{H}}{\partial \theta }\frac{\partial y}{\partial \theta } & ={M\alpha ||e||}_{2}^{2}\\ \frac{\partial y^{H}}{\partial r}\frac{\partial y}{\partial r^{T}} & =MJI\\ \frac{\partial y^{H}}{\partial f}\frac{\partial y}{\partial f^{T}}\; & =\varepsilon \mathrm{diag}(r_{0}{\left(k_{1}\right)}^{2},\cdots ,r_{0}{\left(k_{R}\right)}^{2})\\ \frac{\partial y^{H}}{\partial \theta }\frac{\partial y}{\partial r^{T}} & =0\\ \frac{\partial y^{H}}{\partial \theta }\frac{\partial y}{\partial f^{T}} & =0\\ \frac{\partial y^{H}}{\partial r}\frac{\partial y}{\partial f^{T}} & =\eta \mathrm{diag}(r_{0}\left(k_{1}\right),\cdots ,r_{0}\left(k_{R}\right)) \end{array} \end{array}$$

where $\alpha =\frac{4\pi^{2}}{c^{2}}\sum_{h=0}^{H-1}{\left(u_{h}\cos\left(\theta \right)-v_{h}\sin\left(\theta \right)\right)}^{2}$, $e={\left[k_{1}r_{0}\left(k_{1}\right),\cdots ,k_{R}r_{0}\left(k_{R}\right)\right]}^{T}$, η=-jπ(1+M), $\varepsilon =\left(4\pi^{2}/N^{2}\right){∥u-1∥}_{2}^{2}$. Applying the well-known matrix inverse lemma, the CRB of CSJSR-DoA is:

(C4) $$\begin{array}{c} CRB\left(\theta \right)=\frac{\sigma^{2}}{{2M\alpha ∥e∥}_{2}^{2}} \end{array}$$
